# Effects of Toxic Metal Contamination in the Tri-State Mining District on the Ecological Community and Human Health: A Systematic Review

**DOI:** 10.3390/ijerph17186783

**Published:** 2020-09-17

**Authors:** Hyejoon Park, Keeyoon Noh, Jihyun Jane Min, Christopher Rupar

**Affiliations:** 1 Department of History, Philosophy, and Social Sciences, Pittsburg State University, 1701 S. Broadway, Pittsburg, KS 66762, USA; knoh@pittstate.edu; 2 Thomas Jefferson Independent Day School, 3401 Newman Rd, Joplin, MO 64801, USA; jane.m0820@gmail.com (J.J.M.); crupar@tjeffschool.org (C.R.)

**Keywords:** TSMD, lead, zinc, cadmium, arsenic, PRISMA, ecology, community, health conditions

## Abstract

Although extensive research exists on toxic environments in the Tri-State Mining District (TSMD), there has been a lack of research on how harmful effects in TSMD could affect residents living in those areas. However, quite recently, such research regarding relationships between the health conditions of residents and toxic elements in the TSMD began to grow. The increase of empirical studies means greater complexity of the findings that require a more intricate understanding. To meet the goals of this study, an extensive, systematic review of the literature using PRISMA was conducted. This method resulted in 19 articles that define the harmful effects of the TSMD on the ecology and the physical health of residents. This research found that toxic metals not only negatively impact natural processes in the TSMD environments (fish species reduction, kidney and liver problems, and toxic diet) but also continuously affect the health of residents (high blood Pb and mortality).This study makes a vital contribution building upon the existing outcomes of the correlations between toxic elements in the TSMD areas and the health of residents. Furthermore, conclusions of this study provide updated information to policymakers and health-related professionals by providing adequate and innovative remediations and health-related services in the TSMD.

## 1. Introduction

**Figure 1:**
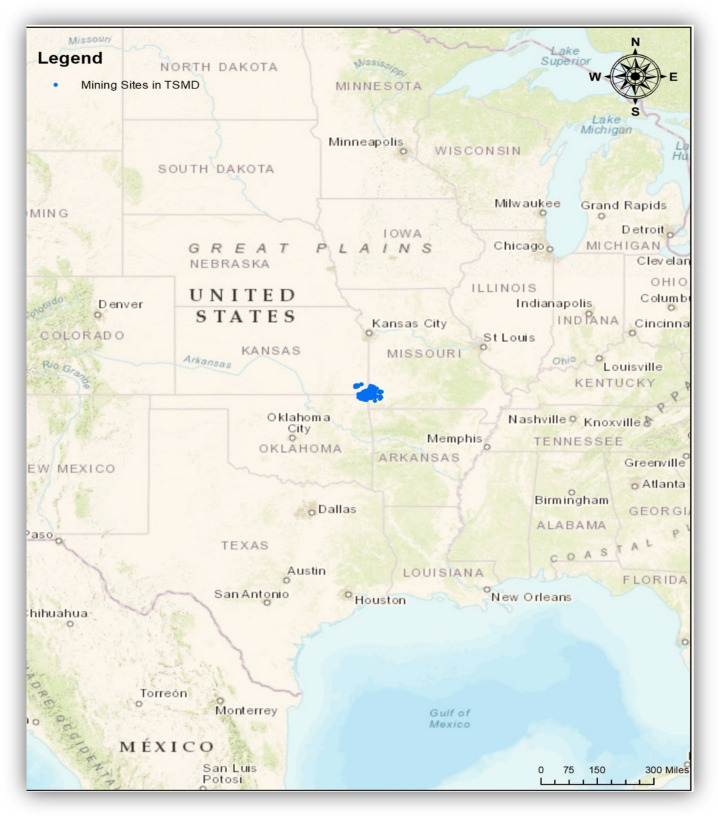
Locations of the Tri-State Mining District (TSMD) in the Unites States. Note: The TSMD is located in the Tri-State area (southeast Kansas, southwest Missouri, and northeast Oklahoma). Blue indicates mine-related sites including mines, mineral deposits, and mineral regions in the TSMD. The geospatial database was obtained from the U.S. Geological Survey (USGS) (Date taken: 29 July 2020). The USGS data are publicly available from https://www.usgs.gov/.

The Tri-State Mining District (hereafter TSMD) of Kansas, Missouri, and Oklahoma in the United States mined lead (Pb) and zinc (Zn) actively from 1850 to 1970 and has been shut down since 1970 [1]. (Figure 1) Even though the mining companies moved out, the TSMD groundwater, soil, and sediments have been contaminated due to the concentration of toxic metal components, such as Pb, Zn, and Cadmium (Cd), in spite of remediation efforts [1,2,3]. Studies regarding the effect of these toxic components on human health (kidney and lung cancer) and ecological systems (e.g., aquatic systems and wild birds) have been increasing for more than a decade. For instance, Beyer et al. [2] found that wild animals, such as northern cardinals and waterfowl living in the TSMD had increased Pb levels, which led to the decrease of red blood cells; Coolon and his colleagues [4] discovered that there has been a harmful impact to mammals living in the TSMD (e.g., reducing biodiversity); and Allert et al. [5] found that aquatic habitats in the TSMD with increased heavy metal levels reduced the biodiversity of the population. In addition, children living in the TSMD had elevated blood Pb levels causing neurological disorders [6], and residents in the TSMD were more likely to have a stroke, chronic kidney disease, hypertension, heart disease, skin cancer, and anemia [7,8,9]. Still many low-income households and ethnic minority people (Native Americans) living in the TSMD earn their living predominantly through agriculture (wheat, sorghum, corn, soybeans, and hay) and pastureland for livestock grazing. They look to enhance their income through hunting and fishing opportunities [10].

Progress has been made by the U.S. Environmental Protection Agency (USEPA) and other stakeholders to remediate the problems of TSMD [3]. Even though the condition of Pb, Cd, and Zn in water, soil, and sediments have improved [3], living creatures, including humans, in the TSMD are still susceptible to the toxic metals. Accordingly, numerous studies have examined habitats, remediations, and toxic components in the TSMD. Unfortunately, very few studies have focused on the TSMD residents’ health conditions, even though many scholars [11,12,13] have found significant relationships between drinking water and physical conditions such as obesity. Therefore, this study addresses how the toxic components lead to degrading the physical conditions of TSMD residents by utilizing systematic review analysis.

For this purpose, first, we described the history and the characteristics of the TSMD, the conditions of habitats in the TSMD, and how toxic components would affect the TSMD ecological system and human bodies in the literature review. Secondly, for the analysis, we utilized Endnote to search and reserve articles relevant to the subject with a diverse database, as well as the Preferred Reporting Items for Systematic Review and Meta Analyses (PRISMA).

Finally, we discussed how our study findings would make a vital contribution building upon the existing outcomes of the correlations between toxic elements coming from the TSMD areas and health conditions of low-income residents by suggesting several recommendations. Additionally, this study outcome will provide updated information for policymakers and public health-related professionals who aim to promote economic and social justice by providing adequate and innovative policies and practices in TSMD rural areas.

## 2. Literature Review

### 2.1. History of the TSMD

The TSMD was initially located in Leadville near Joplin, Missouri, in 1848 and in Granby, Missouri, in 1850. This onset of mining business expanded to Galena, Kansas, in 1876; Aurora, Missouri, in 1886; Peoria, Oklahoma, in 1891; and Commerce, Oklahoma, in 1905 [3]. Mining activity in the TSMD was prosperous between 1880 and 1955. The TSMD, in particular, during this period of time had been known for its leading industry of mining Pb and Zn. The business, however, declined after 1955, and the entire mining industry was closed in the 1970s [3].

The TSMD of Kansas, Missouri, and Oklahoma had produced ore since 1850. However, by the 1970s, ore was depleted, and most mines and smelters shut down. Overall, the TSMD produced 23 million tons of Zn concentrates and four million tons of Pb concentrates [14]. Non-ore waste rock and mill wastes were piled up near production centers in masses called chat piles [15]. Emissions from smelters also accumulated to the metal content of topsoils through fugitive dust and fallout [3]. (Figure 1)

**Figure 2:**
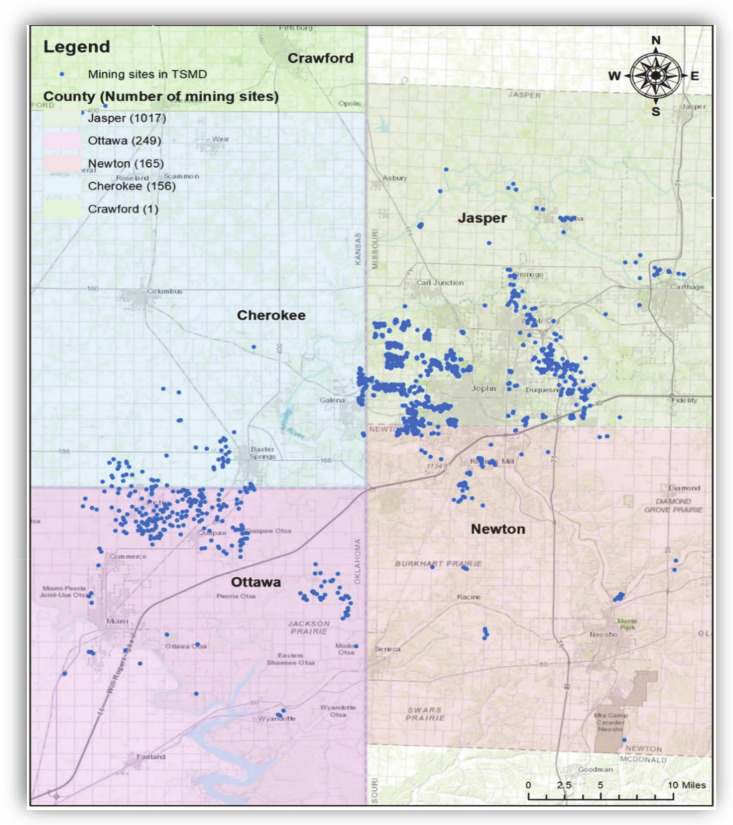
Locations of TSMD Superfund Sites. Note: Blue indicates the mine-related sites in the TSMD. There were 1017 sites in Jasper County, Missouri, 249 sites in Ottawa County, Oklahoma, 165 sites in Newton County, Missouri, and 156 sites in Cherokee County, Kansas. The geospatial database was obtained from the U.S. Geological Survey (USGS) (Date taken: 29 July 2020). The USGS data are publicly available from https://www.usgs.gov/.

There is a large portion of toxic metal areas, which are called “Superfund” sites by the US EPA [16]. Despite remedial actions, tons of waste materials (in particular, Pb, Cd, and Zn), mining shafts, and tunnels are still left in those areas, which in turn contaminated groundwater and soil [3]. Most of all, the components of Pb and Zn are associated with the geographical location of former mining and smelting centers [3,16]. Figure 2 shows the locations and distribution of the mine-related sites in the TSMD Superfund sites.

### 2.2. Characteristics of TSMD Superfund Sites and Their Contamination

In the 1980s, the USEPA designated some areas in the TSMD as Superfund sites—Tar Creek, the area of Picher in “Ottawa County” in Oklahoma, “Cherokee County” in Kansas, and the Oronogo-Duenweg Mining Belt in “Jasper County” in Missouri.

#### 2.2.1. Cherokee County (Kansas) Superfund Site (298 km^2^)

After the mining operation and dewatering pumps were stopped, mines started flooding into a local aquifer. The range of concentration of Cd, Pb, and Zn in these areas was much greater than any other regions (0.6 to 469 mg/kg for Cd, 22 to 7460 mg/kg for Pb, and 100 to 45,000 mg/kg for Zn) [17]. These intensively higher concentrations were detected in mining-affected streams near the Short, Tar, and Spring Branch Creek watersheds [17]. Concentrations exceeding USEPA guidelines cause toxicological effects to aquatic life forms [17]. The U.S. government began to remediate this site in 1986, which aimed to prevent the flow of acidic waters by blocking the aquifer and plugging abandoned wells and mine shafts [3].

#### 2.2.2. Jasper County (Missouri) Superfund Site (498 km^2^)

Jasper county includes Joplin, Webb City, and Carterville. Residential soils in Joplin were primarily impacted by dust and fallout from a smelter that was open until 1970, and mining wastes [3]. Contaminated metallic soil was removed from 2600 properties by 2002, water supply systems improvements were completed in 2007, and 6.06 km^2^ of milling waste were remediated by 2012 [3].

**Figure 3:**
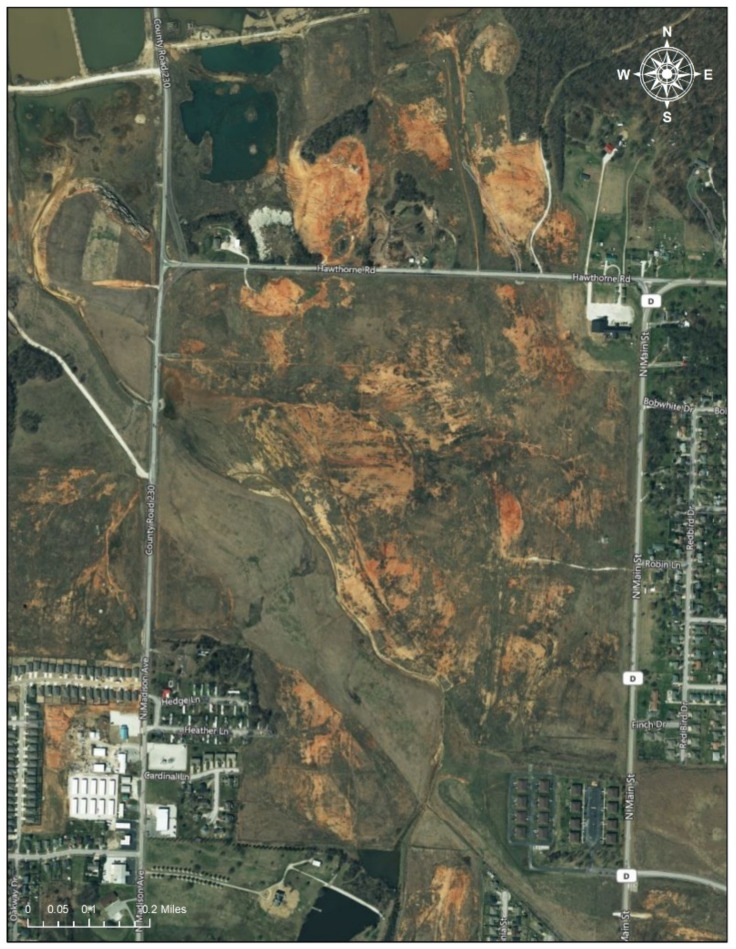
Oronogo-Duenweg Mining Belt Site. Note: The Oronogo-Duenweg Mining Belt Site was one of the major mine waste areas near Joplin, Missouri. The satellite imagery, obtained from Google, shows that the site has left marks (the reddish brown areas) since it was inactive in the 1970s (Date taken: 1 August 2020). The image in the Figure 3 is eligible for the Fair Use of a copyrighted work from Google Map [19].

However, the 2011 tornado (EF-5) brought up the issue of the displacement of contaminated soils throughout the area, and new funding was collected for additional remediation. Consequently, various assessments to detect the dispersion of metals by the tornado in the city of Joplin have been initiated [3,18] (Figure 3).

#### 2.2.3. Tar Creek, Ottawa County (Oklahoma) Superfund Site (104 km^2^)

The Tar Creek Superfund Site covers the cities of Picher, Miami, Commerce, Cardin, and Peoria in Oklahoma. Mining wastes containing Cd, Pb, and Zn were found in 60-m-high piles and sediments of former flotation ponds. Along with the designation of the Superfund site, the USEPA implemented remediation from 1984 to 1986, including plugging wells and constructing dikes to divert water around abandoned mines and collapsed mine shafts3 Water combined with sulfide minerals to become acidic, which dissolved metals [3].

**Figure 4:**
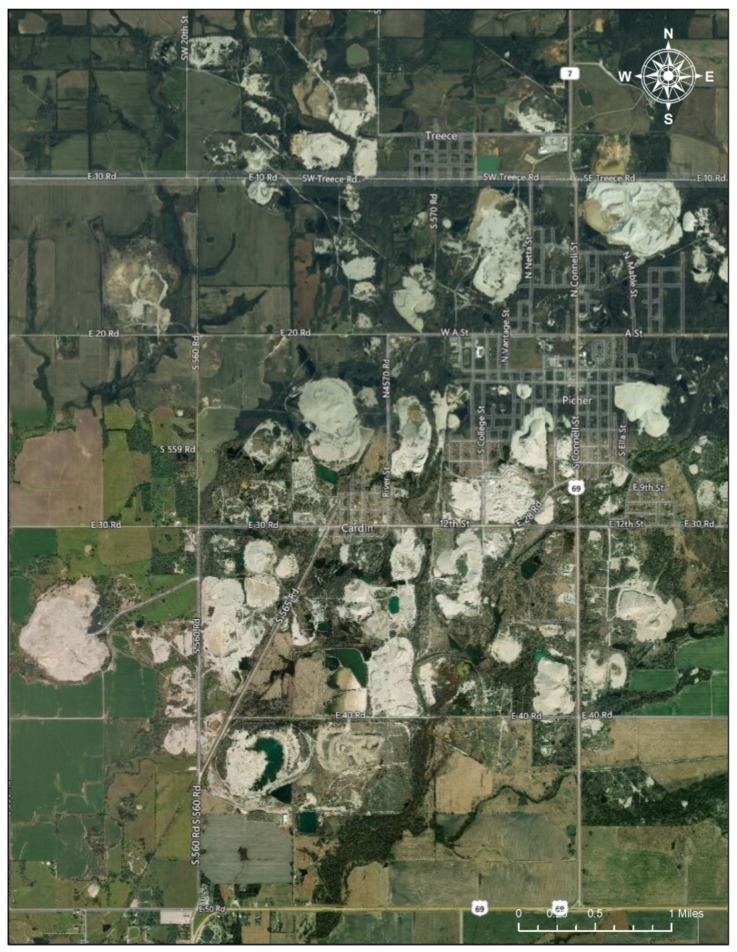
Mining Sites near Treece and Picher. Note: The satellite imagery shows areas with mining sites (the white areas in the picture) clustering near Treece and Picher, Oklahoma, in the Tar Creek Superfund Site (Date taken: 1 August 2020). The image in the Figure 4 is eligible for the Fair Use of a copyrighted work from Google Map [19].

This acidic water contaminated with aqueous metals overflowed into Tar Creek [20]. The remaining chat is widespread in residential areas of Picher [21]. Nine Native Americans tribes—Cherokee, Eastern Shawnee, Miami, Modoc, Ottawa, Peoria, Quapaw, Seneca Cayuga, and Wyandotte—live near the boundaries of the Tar Creek Superfund Site areas and, due to contaminated food and water in these areas, have had to address the environmental health problems [20] (Figure 4).

### 2.3. The Impact of Toxic Metal Components on Ecological Systems

**Figure 5:**
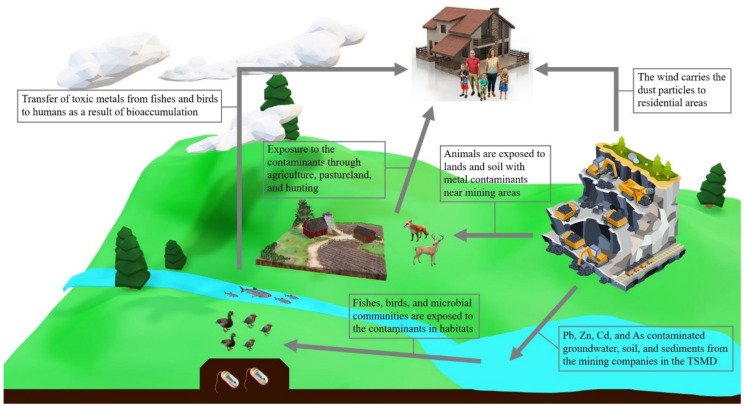
Impacts of Toxic Metals on the Ecosystem. Note: The graphical abstract above shows the impacts of toxic metals on the ecosystem. The toxic metals, such as lead (Pb), zinc (Zn), cadmium (Cd), and arsenic (As), in the wastes from the mining sites and companies in the Tri-State Mining District (TSMD) contaminate the entire ecology from the nature environment to human life; the images in the Figure are eligible for the Fair Use of a copyrighted work [19].

The bioaccumulation of contaminant metals in the TSMD has been detected frequently in various studies. Increased levels of Pb, Cd, and Zn in soils and water in the TSMD severely and negatively impact its environment [3] (Figure 5).

Impacts on wildlife: Areas within and near the mining district have remnants of Cd, Pb, and Zn even after remediation efforts. Unfortunately, these metal contaminants have been harming the living organisms around them. For many wild birds within the TSMD, the metal contaminants have caused serious health effects [2]. These birds may have been exposed to the contaminants through inhalation or ingestion of Pb from chat or plants. The brown thrasher, cardinal, and robin most likely consumed Pb-contaminated soil or food because Pb usually appears on the surface of the soil where birds can accidentally ingest it. Birds can also be exposed to Zn by consumption of the freshwater vascular plants that absorb Zn-contaminated water; aquatic plants can also build up iron and Zn plaque on their roots. Therefore, birds who consume these plants are also ingesting metal contaminants. It is important to note, however, that because contamination throughout the TSMD is sporadic, the average exposure of these wild birds to the contaminants, as well as the concentration of metal within the soil, only give a partial assessment of the factors that are harming the birds [2].

In terms of the metal contaminant Pb, wild bird species—the American robin and other waterfowl in the TSMD—have been harmed by its presence. For instance, American robins, waterfowls, and northern cardinals have increased concentrations of Pb in their tissue, which cause weakened biological functions and indications of poisoning. In addition, the activity of delta-aminolevulinic acid dehydratase (ALAD), Pb-sensitive enzymes, in the red blood cells of these species of birds was reduced by over 50%. This is an indication that these wild birds did not have strong mediators to the toxicity of Pb [22]. The same results could be seen for songbirds, which also had a reduced ALAD activity of less than 10%, which is an indicator of heavy exposure to Pb. Metal contaminants can also be very lethal to wild birds. Many mallards, teals, and pintails have died along the Spring River near Riverton, Kansas, because of Pb poisoning [2].

Within the TSMD, songbirds and swallows had a high concentration level of Cd in their kidneys and livers, and these concentrations are much greater than those birds outside of the TSMD [2]. The waterfowl and trumpeter swan also had Zn in their livers and kidneys. Waterfowls within the district also experienced pancreatitis, the inflation of the pancreas due to the Zn. Overall, the mean metal concentrations of Pb, Cd, and Zn that affected the digestive system of these birds were much higher than their concentrations in wild birds outside of the TSMD with these comparable values (units in milligrams per kg) [2]. In addition, the runoff of metals that were extracted during the mining process contaminated water sources, which caused a decline of fish populations; in particular, the Neosho madtom that lives in these waters is currently listed by the federal government as one of the most severely threatened species of fish [2].

Impacts on microbial communities: Although bacteria are often overlooked, the effect of metal contaminants on microbial communities influences the health of other organisms and the environment. Metal contaminants often decrease bacterial diversity, bacterial biomass, and the richness of an environment. This can cause serious disruptions in the ecosystem by harming the health of other organisms and biochemical elements in soil [4]. Particularly for other organisms, the reduced bacterial diversity and biomass result in less bacterial by-products, which are important for nutrient absorption for animals by assisting the intestinal epithelial cells. Therefore, many rats that live within remediated sites have reduced microbiota diversity, meaning that metal contamination is a factor in their declining health. There is currently no research published specifically on the effects of metal contaminants of the TSMD on bacterial communities and other organisms. However, it can be assumed that the same circumstances in other heavily contaminated sites would occur within the TSMD [4].

### 2.4. The Impact of Toxic Meta Components on Human Health

A study by Zota et al. [13] examined the ways in which children could be exposed to metal contaminants. Within the study, researchers collected yard soil, house dust, and particulate matter of homes near chat sources to test for Zn, Pb, Cd, manganese (Mn), and arsenic (As). They discovered that the dust within the homes had a higher concentration of Zn, Pb, Cd, and As than the dust concentrations discovered in the nearby soil, and Zn had the highest concentration. The metal particles from chat sources can easily enter the home as the wind can carry the particles right outside someone’s home.

Therefore, the metal particles can travel indoors and mix with a house’s dust particles. Even those who do not live near a mining site can be exposed to metal contaminants if they are close to secondary uses of chat. This study by Zota et al. [13], overall, shows that even decades after the TSMD closed, it can still affect various families living within the district. Especially for children who live inside these homes, metals can easily enter their system through ingestion, inhalation, or dermal absorption because many children crawl and frequently put their hands in their mouths [13].

Furthermore, water with Pb contamination has been seriously challenging for utility companies to deal with and affects the potential health of residents who consume the water [11]. There are metal ores present in the shallow aquifers in the TSMD, which are a source of drinking water for residents of the area with private wells [23]. Overall, these studies show there are numerous ways in which residents of the TSMD are highly likely to be exposed to metal contaminants (e.g., Pb poisoning) from past mining activity and end up experiencing adverse health effects. Another study conducted by the Agency for Toxic Substances and Disease Registry in 1995 discovered by examining blood levels of children in Jasper County, MO, that around 14% of children aged 6–71 months had blood Pb levels ≥ 10 μg/dL, compared to no children with elevated blood Pb levels for children in a control group. Average blood Pb levels of adults were also higher than those of people in the control group [16]. A recent study has found that toxic metals can have an effect on obesity rates. In particular, Cd and As influence a person’s reproduction and growth. Cd, an endocrine disrupting chemical (EDC), influences a person’s chances of developing obesity [12]. The study showed that children from seven to nine years old exposed to EDC became overweight or obese in adulthood. For instance, children who had lived in the environment exposed to Cd were likely to become obese/overweight in adulthood [12]. Another study also examined if blood Pb levels influenced obesity in Chinese residents [24]. This study identified that Pb is an endocrine-disrupting chemical, or EDC, meaning that Pb also influences the development of obesity. These studies overall suggest the possibility that the toxic environment of the TSMD could contribute to increased obesity of the people living there.

Faulk et al. [25] discovered that mice, who have similar biological functions to humans, could experience increased body weight and fat as well as greater food intake if they were prenatally exposed to Pb. On the other hand, for humans, this prenatal exposure has been linked with low weight at birth, but these children were more susceptible to becoming obese in the future. In addition, Leasure [26] emphasized that exposure to Pb during the period of gestation could also lead to a child’s increase in body weight. However, after birth, children could continuously be exposed to Pb during childhood, which means they could more easily develop late-onset obesity. This was because Pb was found to alter the hypothalamic-pituitary-adrenal axis, resulting in obesity. Specifically, in a study by Wang et al. [24], there was a positive relationship between high blood Pb levels and a high BMI for women. Another possible explanation for this correlation was that Pb caused oxidative stress and irregularity in a person’s fat metabolism, which could be influencers of obesity. Pb and Cd are some of the common metal contaminants that resulted from the TSMD. Overall, abundant studies indicated that exposure to the toxic contaminants before and after birth could make a person much more susceptible to developing obesity.

Several studies have indicated that toxic heavy metals may play an important role in the development of diabetes mellitus [27,28]. They found a higher incidence of diabetes in arsenic-exposed areas from drinking water, compared to non-exposed areas. Afridi et al. [27] reported that the mean blood Cd concentrations of male non-smoker and smoker diabetic patients were significantly higher than controls. However, a study using the general population in examining the relationship between toxic heavy metals and diabetes found no association between toxic metals and diabetes [29].

## 3. Methods

### 3.1. Search Strategy and Selection Procedure

This study first systematically searched articles from the electronic literature databases Academic Search Premier, American Chemical Society Legacy Archives, PubMed Central (PMC), GreenFILE, JSTOR, and ELSEVIER pertinent to literature, using the terms “TSMD, toxic elements, and negative effects.” Specifically, the search terms were divided into five groups: (1) Tri-State Mining District AND toxicity AND wildlife OR animals OR human health, (2) superfund sites AND toxic contamination AND microbial community OR ecology OR environment, (3) USA AND mining areas AND heavy metal AND physical health OR well-being OR fish OR plants OR birds, (4) USA AND mining AND toxic contamination AND natural system OR plants OR bacteria, and (5) USA AND mining AND arsenic OR zinc OR lead OR cadmium AND environment OR community OR humans.

After locating relevant studies, we exported them into the Covidence software. Once Covidence removed duplicates from the listings, titles, and abstracts were screened to identify potentially related articles by two authors independently. Secondly, these two authors uploaded PDF articles to Covidence to thoroughly review each single article following screen criteria. The third author performed as a mediator when discrepancy took place. Finally, the 19 reviewed articles were created as extraction forms.

### 3.2. Study Selection

**Figure 6:**
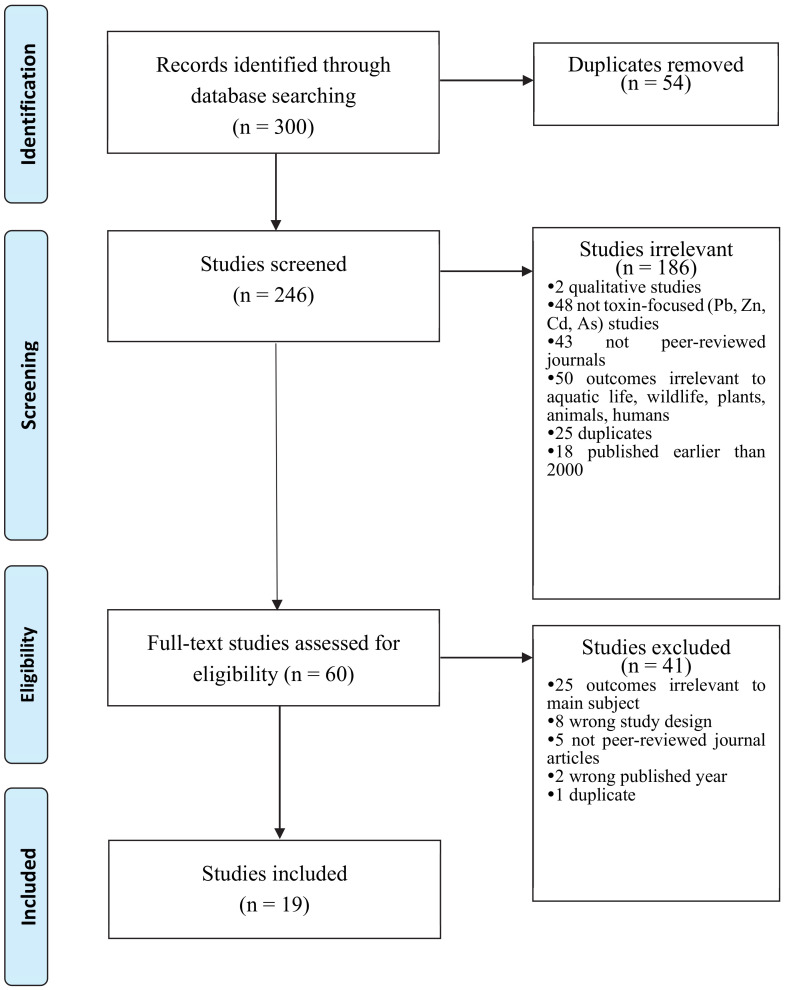
PRISMA Flowchart of Study Selection Process.

Out of 300 articles identified through database searching, 246 studies were screened after removing duplicates (*n* = 54). The screened 246 studies initially excluded several articles based on the following reasons: (a) qualitative studies, (b) studies where the main focus is not toxic/toxin, (c) not peer-reviewed journals, (d) outcomes irrelevant to the main subject (e.g., aquatic life, wildlife, plants, animals, humans, ecology), (e) outdated publication (i.e., earlier than 2000), and (f) duplication. After the excluding process, 60 studies were considered eligible. However, the articles underwent another screening process after detecting irrelevant material. The second process of exclusion criteria was (a) wrong outcomes (e.g., remediation effects), (b) wrong study design (e.g., case studies), (c) not peer-reviewed journal articles, (d) wrong published year, and (e) duplication. As a result, 19 studies remained in the sample. Figure 6 shows the creation of the sample of this study.

Table 1 shows the characteristics of the 19 most eligible studies included in the final sample of this review. For the final process of the sample, the first and second author extracted the following information from each included study: author(s), the purpose of the study, research design/sampling method, key predictor (Independent Variable) measurement, key outcome (Dependent Variable) measurement, sample subject and location, statistical methods, and main findings. 

### 3.3. Quality Assessment

The quality of the included studies was assessed using Standard Quality Assessment Criteria for Evaluating Primary Research Projects from a Variety of Fields designed by Kmet et al. [30]. This assessment was developed using two scoring systems to evaluate the quality of the studies potentially eligible for inclusion in the review [30]. This tool includes 14 items to capture the quality of a study, such as study design, sample, methodology, data analysis, results, and conclusion [31]. Out of two main types of assessment—quantitative and qualitative—we employed a quantitative type for our study assessment. This assessment tool was also used in previous studies for their systematic reviews [31,32,33].

The 14 items were scored by the degree to which the specific criteria were met, yes = 2, partial = 1, and no = 0. Items not applicable to a particular study design were marked “N/A” and were excluded from the calculation of the summary score [30]. The final score of each study was achieved by the formula, ((“yes” × 2) + (“partial” × 1)/28 − not applicable numbers × 2). The quality of the study was expressed by percentage from 0% to 100% [30]. For this study, the first and second authors assessed the quality of each study independently, and any discrepancies were resolved by a third author. The final score in Table 2 represents the consensus between all three authors.

## 4. Results

### 4.1. Risk of Bias

Table 2 presents the result of the risk of bias assessment of the included 19 articles. For two items, “Blinding Investigations” and “Blinding Subjects,” no selected articles received points because of the non-intervention nature of the studies. There was no need, and it was not possible for participants/subjects and researchers to be blinded. Hence all articles received “N/A” for the two categories.

Assessment bias due to method. Some studies such as Coolon [4], Allert et al. [5], Beyer et al. [34], Ettinger [35], Lynch et al. [6], Malcoe et al. [36], Neuberger et al. [37], and Merwe et al. [38] were not able to receive full scores, due to the failure of explanation of the validity of specific samples. However, other studies, Brumbaugh et al. [39], Garvin et al. [40], Schmitt et al. [41], and Struckhoff et al. [42] showed accurately represented population (species) selecting different types of species or selecting them by different locations. Additionally, the study by Yoo and Janz [43] selected different types of female fish (black bullhead and bluegill sunfish) and compared their outcomes seasonally (spring vs. winter).

Assessment bias due to subject: The assessment looks at the clear demographic/characteristic information provided and/or precise description/categorization of participants/samples [30]. Two studies, Beattie et al. [44] and Merwe et al. [38], were not able to obtain the full score since they did not describe the characteristics of their samples to make readers understand the reasons for their subject criteria. On the other hand, Neuberger et al. [37], for instance, described the specific conditions of samples (e.g., health problems) due to the environmental exposures, and Phelps and McBee [45] described the mammal communities’ conditions for the lives of the comparison groups. Additionally, the study by Malcoe et al. [36] hired phlebotomists, interviewers, canvassers, and project coordinators to collect baseline information (e.g., home environment) from the samples’ (children) caregivers.

Assessment bias due to randomization: Due to the characteristics of the study subjects (toxicity, chemistry, and environment), a majority of studies did not conduct randomization except for the two studies conducted by Lynch et al. [6] and Malcoe et al. [36]. The first study clearly addressed that they performed a population-based random sampling as part of a blood lead screening project. By obtaining an overall response rate of over 60%, Lynch et al. [6] collected blood samples from a total of 144 residents out of 550 eligible family representatives of Native American and white households residing within 31 contiguous census blocks in Northeastern Ottawa County, Oklahoma. The study by Malcoe et al. [36] also addressed that their sample consisted of a population-based, representative sample of Native American and white children living within 31 contiguous census block groups in Northeastern Ottawa County, Oklahoma. To recruit eligible families, city blocks were randomly selected within each block group, proportional to the estimated number of households with young children in each block group. At least three visits were made to each residence to determine eligibility, and if there was more than one eligible child in the household, the youngest child was selected to participate.

Furthermore, compared to the rest of the studies that received “N/A,” two studies, Ettinger et al. [35] and Neuberger et al. [37], received a score of zero due to employment of a convenient sampling of the secondary dataset.

Assessment bias due to sample size. This assessment is to see whether the study achieved outcomes from the appropriate sample size. Some studies, Beattie et al. [44], Beyer et al. [2], Beyer et al. [34], and Brumbaugh et al. [39] received a full score because they were able to achieve sizable and comparable samples, other studies, Coolon et al. [4], Allert et al. [5], Struckhoff et al. [42], and Yoo and Janz [43] consisted of a small sample size and other studies; Besser et al. [46], Neuberger et al. [46], and Schmitt et al. [41] showed unbalanced sample group sizes for comparing outcomes.

Assessment bias due to analytic methods. A majority of the selected studies employed advanced and proper analytic methods except for the study by Hays and Mcbee [47], which mostly presented least-square means and relative frequency.

Assessment bias due to variance. A majority of the studies presented an estimate of variance (e.g., confidence intervals, standard errors, and standard deviation) to explain the main results and outcomes except for a study by Garvin et al. [40], which only addressed the sample group differences using ANOVA (2-tailed).

### 4.2. Characteristics of Included Studies

#### 4.2.1. TSMD Toxic Effects on Plants

Two studies—Garvin et al. [40] and Struckhoff et al. [42] did research about the correlations between metal concentrations (Cd, Pb, and Zn) and plants in the TSMD. Specifically, the study by Garvin et al. [40] focusing on 36 species of edible plants and soil from floodplain areas in southwestern Missouri (MO), southeastern Kansas (KS), and northeastern Oklahoma (OK), found that there was a significantly positive association between metal concentrations in plant tissues and soil, and a significant difference in metal concentration distribution between reference plants and impacted plant samples. In particular, most plants included a higher degree of exceedance of Cd and Pb.

The study by Struckhoff et al. [42] used two standard floristic quality measures, mean coefficient of conservatism (Mean C) and floristic quality index (FQI), and examined the concentration of Pb and Zn, soil nutrients, other soil characteristics, and plant communities near mine waste and a Pb smelter in two subregions known as the Old Lead Belt and the Viburnum Trend in the Southeast Missouri mining district (SEMO). They also found that exotic species had the highest Pb concentration (3464 mg/kg) and second-highest Zn concentration (851 mg/kg), while Pb and Zn soil concentration had the highest correlation values. Their findings of lower Mean C and FQI, as well as abundance of non-native species with higher Pb and Zn soil concentrations, supported the adverse effects of elevated metals concentrations on plant community composition and structure [42].

#### 4.2.2. TSMD Toxic Effects on Aquatic Life

We found several articles that examined the effects of TSMD toxic metals on living creatures in the water. First, Allert et al. [5] examined the characteristics of physical habitat and water quality, focusing on toxic metals (Pb, Zn, and Cd) and crayfish. By collecting subsamples of water, sediments, detritus, and crayfish from three metal-contaminated sites in Spring River of southwestern MO and southeastern KS, they found that there were differences in groups of sites (reference, mining, and downstream), there were significant correlations between surface water toxic units and aquatic biota, and toxic metals in crayfish at several sites exceeded concentrations considered harmful to carnivorous wildlife. In addition, Schmitt et al. [48] and Brumbaugh et al. [39] also found a similar outcome that Pb, Zn, and Cd in fish in TSMD rivers were high compared to those in reference sites. Additionally, by testing the blood and livers of their study samples (carp, bass, and catfish), Brumbaugh et al. [39] highlighted that for Zn, differences among sites for both blood and carcass were significant between catfish and bass and between bass and crappie. There were also significant differences among sites for Pb in both blood and carcass.

Two studies—Schmitt et al. [41] as well as Yoo and Janz [43] discovered a direct biochemical effect of Pb, Cd, and Zn on fish living in the TSMD water. First, Schmitt et al. [41] found that delta-aminolevulinic acid dehydratase (ALAD) activity in catfish was more sensitive to blood Pb than in the other species, and catfish from TSMD sites had more problems with enzyme activity than those in reference sites. Overall, Pb was both bioavailable and active biochemically in the Spring–Neosho River system. Secondly, Yoo and Janz [43] observed that aqueous concentrations of Cd and Zn and liver concentrations of Cd and Zn in their study samples, bluegill sunfish, and black bullhead, were significantly greater at Tar Creek compared to the reference site, and the 70-kDa stress protein family (HSP70) expression was consistently elevated in the head kidney of both fish species at Tar Creek compared to fish collected from the reference creek. However, there were no consistent differences observing HSP70 expression in liver, gill, or ovarian tissues between sites.

#### 4.2.3. TSMD Toxic Effects on Wildlife Animals

Five articles, Beyer et al. [2], Beyer et al. [34], Hays and McBee [47], Phelps and McBee [45], and Merwe et al. [38] identified TSMD toxic exposure as relevant to animals Observing wild birds (American robins, cardinals, and waterfowl), Beyer et al. [2] uncovered that Pb tissue concentrations of their study samples from chat environments had increased Pb tissue concentrations (*p* < 0.05) compared to those of reference site birds; mean activities of Pb-sensitive ALAD were decreased by >50% in the red blood cells of these sample birds (*p* < 0.05); Zn concentrations in the liver and kidneys of waterfowl from chat environments were significantly higher (*p* < 0.05) than reference birds; and the increased Zn concentrations in the chat appeared to cause pancreatitis in waterfowl.

Another study from Beyer [34] discovered that earthworms from Southeast MO contributed to high Pb exposure in songbirds which prey on soil organisms. Mean tissue Pb concentrations in songbirds from the sites were greater (*p* < 0.05) than those in songbirds in the reference sites by factors of 8 in blood, 13 in the liver, and 23 in the kidney, and mean activity of the enzyme ALAD in red blood cells was lower by 58–82% in songbirds from the mining sites. Examining bird species (Canada Geese), Merwe et al. [38] detected similar outcomes with toxic metals. Adverse effects associated with Zn poisoning were found from the examination of pancreas tissue, and elevated tissue Pb concentrations and inhibited blood ALAD enzyme activities were consistently found in birds from the TSMD.

Finally, looking at the ecological characteristics of small mammal communities (white-footed mouse in Tar Creek Superfund Site), Phelps and McBee [45] found that the Tar Creek Superfund Site has experienced reduced species diversity, including richness and evenness compared to the non-mining sites. Additionally, species composition was different between contaminated sites and reference sites, as a detrended correspondence analysis indicated that, in terms of species diversity, contaminated sites were quite similar to each other but had fewer similarities when compared to either reference.

#### 4.2.4. TSMD Toxic Effects on Microbial Communities

The two studies by Beattie et al. [44] and Coolon et al. [4] examined bacteria to understand the microbial community structure in the TSMD. First, analyzing 16S r(ribosomal)RNA gene sequences and q(quantitative)PCR of bacteria and archaea, Beattie et al. [44] discovered that bacteria were negatively and significantly correlated with Pb, Cd, Zn, and Mg levels. However, archaea were only significantly and positively correlated with pH. Illumina sequencing of 16S rRNA genes showed significant differences in microbial communities near chat environments and west transect samples, and soil chemistry with community structure indicated that Al, Pb, Cd, and Zn significantly impacted community composition and distribution of individual operational taxonomic units (OTUs). Additionally, Coolon et al. [4] found that the assemblage of bacterial communities differed with respect to contamination in remediated sites. Both studies emphasized that it is vital to provide ecosystem services to chat environments and understand the effects of long-term heavy metal contamination on microbial populations.

#### 4.2.5. TSMD Effects on Human Health

Four studies, Ettinger et al. [35], Lynch et al. [6], Malcoe et al. [36], and Neuberger et al. [37], observed the effect of TSMD toxic contaminations on human health. First, using convenient sampling—recruiting 532 pregnant women from a health center near the Tar Creek Superfund Site—Ettinger et al. [35] discovered that blood As concentrations ranged from 0.2 to 24.1 ug/L (ppb) and in hair, it ranged from 1.1 to 724.4 ng/g (ppb); impaired glucose tolerance was observed in 11.9% of women when all participants had the glucose screen test; and after controlling for covariates, women in the highest quartile of blood As exposure had 2.8 higher odds of impaired glucose tolerance test (GTT) than women in the lowest quartile of exposure (95% CI, 1.1–6.9) (*p* = 0.008).

Examining blood lead concentrations (BPbs) from 245 residents in the TSMD, Lynch et al. [6] and Malcoe et al. [36] also found that BPbs were significantly associated with floor dust and yard soil. Malcoe et al. [36] additionally discovered that soil Pb levels and poverty were strongly correlated (*p* = 0.005). All three studies supported the result of the study by Neuberger et al. [37] that the health of the TSMD residents, specifically mortality related to stroke and heart disease, is still affected by ongoing heavy metal exposure even after remediation involvement, and that the presence of toxic concentrations in the TSMD should not be ignored.

## 5. Discussion

In this systematic review, we evaluated 19 studies on the effects of toxic elements in the TSMD on the ecological community and physical health of residents. Harmful metal components from the TSMD mining sites which mined Pb, Zn, Cd, and As were found to affect the surrounding environments and residents negatively through both water and soil. Despite the remediation effort by the U.S. government, we were still able to find the prevalence of harmful effects of the metal components produced from the mining sites. Fifteen studies among the 19 studies in this review focused on the results of toxic metal contaminants on the ecological community, such as plants, aquatic life, wildlife animals, and microbial communities. In all of these studies, heavy-toxic metals were concentrated in natural habitats and the internal organs of animals themselves. The effects of the heavy-toxic metal contaminants, needless to say, are harmful to biodiversity. Their harmful effects on human health should not be overlooked either.

Toxic metals, such as Pb, Zn, and Cd, in mine wastes have been found to environmentally travel between primary sources and other ecological communities. This environmental mobility of toxic metals makes them pose a considerable risk to human health [20,49]. In addition to the included studies in this review, other studies also found that blood Pb levels in children who lived near the toxic metal exposed areas (i.e., the TSMD) exceeded the maximum level recommended by the Center for Disease Control [50,51,52,53].

The TSMD was one of the leading producers of heavy metals in the country. The smelting process in the TSMD left byproducts called chat and slag. Chat can easily harm the local soil along with slag. The soil was not only affected by this waste but also experienced sinkholes that resulted from the mining process. After discovering the high concentrations of Pb in the TSMD, the USEPA recognized the Pb poisoning of local residents. However, the remediation efforts were not completely successful as the mines continue to harm the environment to this day [23]. For instance, many piles of chat remain in Oklahoma, particularly in Tar Creek (south of Picher, OK). The runoff of these piles of waste and mine-water discharge harm the environment. Jasper County, MO, is also experiencing poor water quality as a result of seepage, metal particles traveling to the water, and mining runoff [23]. Other water sources such as watersheds in the TSMD are contaminated with heavy metals because groundwater filtered those metals into the watersheds. However, in general, a high concentration of >1000 ppm of metals was discovered in soil and water sources in areas within the TSMD. It is important to note, however, that the metal contaminants are not just a threat to the local area as contaminants can spread at least 20 km from their original location [44]. In addition, despite remediation efforts, metal contamination continues to affect the local environment, wildlife, and daily life of residents in the TSMD.

This systematic review, however, has some limitations. First, although we rigorously employed a broad search strategy to find related journal articles on this subject matter, there were only a few published studies on the effects of metal contamination in the TSMD on the ecological community and human health. This may be due to a relatively limited number of databases that were available to us for potentially eligible studies.

Second, to assess the included studies, we relied on a single quality assessment tool, Standard Quality Assessment Criteria for Evaluating Primary Research Projects from a Variety of Fields [30] instead of multiple tools. We were not able to find other quality assessment tools that could be universally applied to studies with a lack of homogeneity in terms of study outcomes and designs.

The third limitation is also related to the issue with a lack of homogeneity in study outcomes and designs. It was difficult to synthesize the included studies in this review for a meta-analysis because each of the included studies in this review had an inconsistent study outcome and subject. As seen in the results, the number of included studies for each area of its effects (i.e., plants, aquatic life, wild animals, etc.) ranged between two and five. Conducting a meta-analysis on this small number of studies would not produce meaningful and reliable results.

Fourth, although there have been studies on the effects of heavy toxic metals on the insect community which found that insects were good bioindicators of metal pollution [54,55], no studies on the insect community were included in our review. We could not find such studies from our database searching process, perhaps because such studies were conducted outside the TSMD.

## 6. Recommendations

There are possible and cost-effective remediations recommended by many scholars. The first one is recovering the contaminated areas to reuse and recycle the mining wastes [56]. For example, repurposing mine wastes might produce economic benefits while addressing environmental concerns. Waste rocks can be used as filling for mine shafts and cavities, and benign tailings can be mixed with cement to cover less benign tailings [56]. Rock waste can also be used for pavement, buildings, and other structures through mineralogy [3].

Second, phytoremediation utilization can be practical. This assists in bioaccumulated plant revival through roots, stems, or shoots [57]. Another possible solution is the application of the methodology used in Oklahoma to other Superfund sites. The comprehensive remediation plan for Oklahoma consists of hydrology, the reactivity of contaminants, and bedrock geology. Since the hydrology and geology of Oklahoma and the TSMD are similar, it will be an evidence-based methodology that can be safely and economically utilized in other parts of TSMD areas [3].

Even though several studies relevant to humans’ physical health are smaller than other study foci, it is obvious that the recommended remediation would be not only helpful for habitats in the TSMD environment but also effectively boost the physical well-being of residents in those areas. Additionally, public health policymakers need to make careful applications for the overall health of low-income TSMD households in terms of repaving playground sand or surface materials in play areas, avoiding the use of mine waste piles for the construction of residential homes or public use areas like parks, replacing fill material that comes in contact with free-standing water or with surface water, and using an agricultural soil amendment to adjust soil alkalinity [23]. Additionally, providing regular health checkups by community clinics or health care providers to TSMD residents would be beneficial to prevent the acceleration of health problems related to toxic materials.

## 7. Conclusions

Despite the limitations of the systematic review process that we had, this review clearly shows that the local flora, fauna, and residents of the TSMD are still suffering from the lingering effects of the area’s mining legacy. While remediation efforts by the government have made some progress, they have not gone nearly far enough to ensure the well-being of the people and environment of the region.

One possible impediment to remediation is the fact that a significant portion of the area lies within a karst terrain [58]. The USEPA uses excavation of contaminated chat and sediment followed by disposal in subsidence pits as its preferred remediation strategy in the area [23]. The excavation and disposal technique relies on the isolation of contaminants within the pit to prevent them from entering nearby aquifers. If these subsidence pits are part of a karst topography, the porous and fractured nature of the karst would render this method ineffective. A study of a similar superfund site in Puerto Rico showed that the fractures and conduits within the karst aquifer were responsible for spreading contaminants to nearby streams, wells, and wetlands [59]. In fact, most of the case studies that the USEPA categorizes as impossible to remediate, or technical impracticability, were in areas of karst terrain [60].

Overall, this review provides details about the mechanisms through which heavy metal contaminants from mining industry waste are dispersed and absorbed into the surrounding soil and water, the effects of those metal contaminants on microbe, fish, plant, and bird populations, and the increased health risks to lower-income children and adults living in the area. Policymakers can use the data presented here to influence the decision-making process to show that more remediation needs to be conducted. Furthermore, this review provides several techniques that can be implemented to make sure the land and people no longer have to be impacted by an industry that abandoned the area decades ago.

## Figures and Tables

**Table 1:** Characteristics of the Studies on the Effects of Toxic Contaminations in the TSMD on the Ecological Communities and Human Health.

	Study	Purpose	Research Design/Sampling Method	Key Predictor Variable (IV), Measurement	Key Outcome Variables (DV), Measurement	Sample (Subject/Location)	Statistical Methods	Findings
1	Allert et al. (2012)	Characterize physical habitat and water quality; evaluate the potential effects of metals in crayfish and carnivorous wildlife.	Quantitative; comparisons in different sites (reference, mining, downstream).	Pb, Zn, and Cd in surface water, sediment, detritus, and crayfish.	The concentration of Pb, Zn, and Cd.	*n* = 24/site. Subsamples of water, sediments, detritus, and crayfish from 3 metal-contaminated sites in Spring River of southwestern Missouri (MO) and southeastern Kansas (KS).	Nested analysis of variance (ANOVA) for finding group differences of areas with site considered a fixed effect; Linear regression using PROC REG was used for crayfish densities.	1. Mean densities of crayfish at mining sites were lower than reference sites. 2. Mean concentrations of metal materials were significantly correlated and greater at mining and downstream sites than at reference sites. 3. Sediment probable-effects quotients and surface water toxic units were significantly correlated, indicating the risk of toxicity to aquatic biota at several sites. 4. Metals concentrations in crayfish at several sites exceeded concentrations considered toxic to carnivorous wildlife.
2	Beattie et al. (2018)	Understand changes in microbial community structures due to regional metals contamination (Pb, Zn, Cd, AI, and Mg).	Quantitative; topsoil samples were collected using an ethanol-cleaned metal hand spade at intervals of 0.32 km in each of the cardinal directions (*n* = 100) extending from and directly from mine tailings in chat piles.	Soil pH, moisture, and heavy-metal concentrations of Topsoil samples.	16S ribosomal RNA gene sequences and quantitative PCR calculations of Bacteria and Archaea.	Topsoil samples (*n* = 111; 10 cm depth) were collected within an 8.05 km radius of Picher in Ottawa County, Oklahoma (OK).	Analyzing concentrations of 20 metals with EPA method using an Inductively Coupled Plasma Optical Emission Spectrometer (ICP-OES).	1. Bacteria were negatively and significantly correlated with Pb, Cd, Zn, and Mg. 2. Archaea was only significantly and positively correlated with pH. 3. There were significantly different microbial communities in chat and west transect samples. 4. There were significantly impacted toxic components and distribution of individual Operational Taxonomic Unit (OUT).
3	Besser et al. (2015)	Examine chronic effects of sediment toxicity on freshwater mussels’ survival, growth, and biomass (also known as, amphipod toxicity test).	Longitudinal and quantitative; in 2006, sediment was collected using a Petit Birge-Ekman grab sampler. 2 precleaned 20-L polyethylene sample buckets were filled one third with site water, and a benthos wash bucket with stainless steel screen bottom was placed inside a sample bucket; Juvenile amphipods, midge larvae, larval mussels	Toxic materials in sediments.	Survival, growth (mean weight), biomass (total weight per replicate) for each species.	Tri-State (*n* = 58–65), Southeast MO (*n* = 16). Every 20 L of bulk sediments and porewater (wet sieved to <2 mm particle diameter) were collected from Tri-State sites, southeast MO, and reference sites; juvenile amphipods, midge larvae, and larval mussels	Toxicity test using ANOVA, EC 10 (for metal mixture), and principal components analysis to evaluate relationships among metal concentrations, sediment characteristics, and responses in toxicity tests.	1. The frequency of highly toxic responses in Tri-State sediments was greater for amphipod survival (25% of samples), midge biomass (20%), and mussel survival (14%). 2.The frequency of highly toxic responses in southeast Missouri sediments, the frequency of highly toxic was greater for mussel biomass (25%), and amphipod biomass (13%). 3. Thresholds for metal toxicity to mussels were lower for southeast MO sediments than for Tri-State sediments. 4. Southeast MO sites with toxic sediments had 2 or fewer live mussel taxa, compared with 7 to 26 taxa at reference sites.
4	Beyer et al. (2005)	Determine if the habitat of the TSMD has been contaminated by the espousal of toxic concentrations of metals.	Quantitative; wild birds were selected in chat piles.	Toxic materials in tissues and blood of sample birds.	Biological functions and external signs of poisoning.	An experimental group (13 species) were collected from the TSMD area from December 2000 and August 2001, and a reference group (the same species) was collected from uncontaminated sites (Neosho Wildlife Area, St. Paul, KS, and cliffs in Chestertown, Maryland (MD).	Toxic materials from samples of tissues were quantified by ICPMS/ICP-ES; blood samples were analyzed for ALAD activity.	1. American robins, northern cardinals, and waterfowl had higher Pb tissue concentrations (*p* < 0.05) than Pb tissue concentrations from reference birds. 2. Mean activities of the Pb-sensitive enzyme delta-aminolaevulinic acid dehydratase (ALAD) were decreased by >50% in red blood cells in these birds (*p* < 0.05). 3. Some birds had tissue concentrations of Pb that have been associated with impaired biological functions and external signs of poisoning. 4. Zn in the liver and kidney of waterfowl were significantly higher (*p* < 0.05) than reference birds.
5	Beyer et al. (2013)	Estimate the potential exposure of songbirds to Pb in southeastern MO.	Quantitative; earthworms, soil, 34 adult, and juvenile songbirds collected from southeastern MO were collected, reference songbirds remote from Pb mining; one composite sample of eight soil cores was collected at each site.	Earthworms associated with Pb concentrations of soil.	Mean tissue Pb concentrations in songbirds.	All songbirds (reference = 39, mining site = 34). Birds were captured at least 4 weeks after spring migration.	Blood (1% of body weight) of birds was taken with a mic needle. Red-blood cell ALAD activity was measured. The soil samples from the sites were quantified for detecting toxic materials; the tissue metal concentrations and ALAD activities in songbirds from mining sites were compared with reference birds using ANOVA.	1. Mean tissue Pb concentrations in songbirds from the contaminated areas were greater (*p* < 0.05) than those in songbirds from the reference site. 2. Out of the total birds (*n* = 34), 22 had hepatic Pb concentrations consistent with adverse physiological effects, 3 with systemic toxic effects, and 4 with life-threatening toxic effects. 3. Acid-fast renal intranuclear inclusion bodies, indicative of Pb poising, were detected in kidneys from 2. 4. Mean activity of the ALAD in the red blood cell, a well-established bioindicator of Pb in birds, was decreased by 58–82% in songbirds from the mining sites.
6	Brumbaugh et al. (2005)	Assess the surface-and groundwater contamination in the Spring River Neosho River (SR-NR) system of northeastern OK.	Quantitative: 74 fish from 6 locations in the SR–NR system were collected (e.g., catfish, bass, and white crappie).	Pb, Cd, and Zn in tissues, blood, and liver of sample fish.	High levels of toxic contaminations in fish.	Sample size is 74. Sample fish were collected from TSMD-affected portions of the SR and NR in northeastern OK; 4 specimens of each of three primary species were targeted at each OK site (i.e., common carp, largemouth bass, and channel catfish).	The ICP-MS was programmed to determine Zn, Cd, and Pb; for each variable, species-station arithmetic means and standard errors were computed; ANOVA was conducted for each variable; a one-way ANOVA was considered for testing for differences among collection sites; Fisher’s protected LSD was used for differences among individual sites.	1. Cd and Pb in carp and catfish from OK and Pb in carp and catfish from MO were elevated. 2. Zn in bass and crappie were low. 3. Variability was high for Cd in all three tissues of crap; differences between sites were significant only for blood, even though the mean liver was 100-fold greater than those in blood. 4. Blood concentrations of Cd and Pb were positively correlated with the concentration of the same element in crap and catfish, and the corresponding multiple regression models were highly significant.
7	Coolon et al. (2010)	Examine the effect of residual contamination on rodent and the microbial communities at remediated sites in the TSMD.	Quantitative; using 25 trap stations per site, trapped a representation of the small mammal community; tapped 10 *Peromyscus. maniculatus* and 6 *Permyscus. leucopus,* euthanized and necropsied in the field.	Heavy metal exposure.	Bacterial community diversity (soil bacteria).	10 rodents’ pair (2 species) from the TSMD and non-TSMD.	Massively parallel sequencing (MPS) technologies to sequence bacterial 16S DNA amplified from the soil and mouse intestines.	1. Rodents on the remediated site had reduced body mass, smaller body size and lower body fat than animals on reference sites. 2. Bacterial communities in both the soil and *Peromyscus* spp. Gastrointestinal tracts had no difference in diversity between reference and remediated sites, but assemblages differed in response to contamination.
8	Ettinger et al. (2009)	The association between the level of arsenic and impaired glucose tolerance.	Correlational and quantitative; screening pregnant women during prenatal visits at the hospital in Ottawa county.	Toxic elements in blood and hair.	Blood glucose.	532 pregnant women/The Tar Creek Superfund Site.	Univariate, bivariate, and logistic regression analyses.	1. The concentration of arsenic was found between 0.2 and 24.1 ug/L (ppb) and 1.1 to 724.4 ng/g (ppb) in blood and hair, respectively. 2. One-hour glucose levels ranged from 40 to 284 mg/dL; impaired glucose tolerance was observed in 11.9% of women as using standard screening criterion (>140 mg/dL). 3. Women in the highest quartile of blood arsenic exposure had 2.8 higher odds of impaired GTT than women in the lowest quartile of exposure.
9	Garvin et al. (2018)	Determine metal concentrations in consumed plant species in the TSMD.	Quantitative; collect 36 species of edible plants and soil from floodplain areas, plants from reference sites.	Cd, Pb, and Zn in plants.	Cd, Pb, and Zn in soil.	The sample size is 210; 36 species edible plants from various floodplain areas for tribal communities in the TSMD soil samples.	ICP-MS; Spearman rank correlation.	1. A significantly positive correlation between metal concentrations in plant tissues and soil. 2. A significant difference in metal concentration distribution between reference and impacted plant samples.
10	Hays & McBee (2010)	Investigate the effect of Pb and Zn on the ecology of Red-Eared Slider Turtles.	Quantitative; out of 327 turtles, 293 individuals were used to determine sex ratios, SDI, and average sizes.	Toxic elements in the TSMD.	Body size, sex ratios, sexual dimorphism indices, and recapture and survival rates.	293 *Trachemys scripta* (165 males, 128 females) from Tar Creek Superfund Site (TCSFS) and reference cites (Sequoyah National Wildlife Refuge, Lake Carl Blackwell).	Chi-square test, goodness-of-fit tests in RELEASE, saturated global model, adjusted Akaike’s Information Criterion.	1. Sex ratios were female-biased at TCSFS and Lake Carl Blackwell and male-biased at Sequoyah National Wildlife Refuge. 2. Degree of sexual size dimorphism differed among the 3 sites. 3. Male turtle was significantly larger at Lake Carl Blackwell than at reference sites. 4. Females from TCSFS were significantly larger than females from Lake Carl Blackwell. 5. Survival and recaptures rates did not differ significantly in two different areas.
11	Lynch et al. (2000)	Examine the independent contributions of various lead sources to elevate blood lead levels in area children.	Quantitative; (a representative random sample of Native American and white households residing within the study area).	Level of lead in sources (from the soil, dust, and paint). The paint was measured using protocols of the U.S. Department of Housing and Urban Development and the U.S. Environmental Protection Agency.	Elevated blood lead level measured by micrograms per deciliter (10 μg/dL).	*n* = 244, Native American and white residents within 31 contiguous census blocks in northeastern Ottawa County, Oklahoma.	Logistic regression using SAS and EpiInfo to estimate associations between environmental exposures and elevated blood lead levels.	1. Floor dust, yard soil, interior paints, and location of residence were independently associated with elevated blood lead levels.
12	Malcoe et al. (2002)	Examine the effects of lead sources on blood lead concentrations (BPbs) in rural children.	Quantitative; (a population-based, representative sample of Native American and white children in the study area).	Paint indices measured as Index Value = (*Pb Concentration*) × (*Size of Sample Area*) × (*Deterioration Value*). Socioeconomic and behavioral measures were used from interview data.	Blood lead concentrations measured by micrograms per deciliter (10 μg/dL).	*n* = 224, Native American and white children in Ottawa County, Oklahoma.	Non-parametric Wilcoxon rank-sum test and multiple linear regression using SAS for BPb variability.	1. Soil and dust lead derived largely from mining waste pose a health hazard to Native American and white children, and that current residential dust lead standards are insufficient to protect children adequately. 2. Poor children were especially vulnerable to lead exposures suggests that residential standards should consider interactions among socioeconomic conditions and lead sources if environmental justice is to be achieved.
13	Neuberger et al. (2009)	Examine the potential impact of exposure to heavy metals and health problems.	Quantitative; (secondary data obtained from the Oklahoma State Department of Health).	Geographic comparisons (exposed areas of Ottawa County vs. unexposed areas).	Mortality outcomes (lung cancer, Tuberculosis, Bronchitis, emphysema, asthma, kidney disease, hypertension, stroke, and heart disease), health outcomes in the first year of life (low birth weight, infant mortality, and infant mortality excluding infectious diseases).	*n* = not clear, residents at or near the Superfund site in Ottawa County, Oklahoma.	Standardized Mortality Ratio (SMR) (observed versus expected mortality calculation by ratio) and a Poisson model.	1. Excess mortality was found for stroke and heart disease when comparing the exposed County to the state but not when comparing the exposed cities to the nonexposed rest of the County.
14	Phelps & McBee (2009)	Determine ecological characteristics of small mammal communities inhabiting a heavy metal contaminated site, Tar Creek Superfund Site, compared to reference sites located in northeastern Oklahoma over a 2-year timeframe.	Quantitative; (small mammal communities were sampled at two locations within Tar Creek Superfund Site (TCSFS) and two uncontaminated reference sites).	Geographic comparisons (two locations within TCSFS vs. two uncontaminated sites).	Species diversity measured using Simpson’s diversity index, rank abundance analysis measured using Southwood and Henderson (2000)’s method, and differences in community composition among sites evaluated using detrended correspondence analysis (Ter Braak and Smilauer, 2002).	*n* = not clear, small mammal communities within TCSFS and outside the immediate area surrounding TCSFS.	Simpson’s diversity index, rank abundance analysis, and detrended correspondence analysis with GANOCO.	1. Tar Creek Superfund. The site had reduced species diversity, including richness and evenness, compared to the reference sites. 2. Species composition was different between contaminated sites and reference sites, as evidenced by detrended correspondence analysis, with contaminated sites being more similar to each other than to either reference site. 3. No direct link between site contamination and disparities among most ecological characteristics could be established.
15	Schmitt et al. (2005)	Examine biochemical effects of lead, zinc, and cadmium from mining on fish in the TSMD.	Quantitative; (fish representing six species were collected from six sites on the Spring and Neosho Rivers, and additional samples from the Big River).	Concentrations of Zn, Cd, Pb, and iron in the blood of six species of fish.	d-aminolevulinic acid dehydratase (ALAD) activity, and Hb-adjusted ALAD activity (ALAD/Hb) in the blood of fishes.	*n* = 74, fish representing six species were collected from six sites on the Spring and Neosho Rivers, and additional samples from the Big River.	One-way ANOVA using the site as a fixed effect and stepwise multiple linear regression using SAS.	1. ALAD activity was inhibited by more than 50% in catfish from several TSMD sites, which is evidence that Pb is both bioavailable, and active biochemically.
16	Struckhoff et al. (2013)	Examine the effects of mining-associated lead and zinc soil contamination on native floristic quality.	Quantitative; (plant communities were sampled in three strata.	Soil concentrations of lead and zinc measured as mg/kg.	Mean C and Floristic Quality Index (FQI) measured using Floristic Quality Assessment methods (Swink and Wilhelm, 1994).	*n* = 76 (Mean C = 38, FQI = 38), plant communities in the southeast Missouri Mining District.	Least-square regression trees to identify variables that best explain variation in Mean C and FQI and univariate regression.	1. Significant negative relationships between both Mean C and FQI.
17	Merwe et al. (2011)	Assess the presence of preclinical lesions, metals accumulation in tissues, and physiologic markers of adverse health effects associated with metals exposure on Canada geese.	Quantitative; (birds were collected by the U.S. Fish and Wildlife Service personnel).	Geographic locations (four mine waste-exposed sites vs. a reference site).	Lead and zinc concentrations in bird tissues.	*n* = 28, Canada geese from the TSMD and the reference site at a farm pond 1.6 km northeast of Neosho State Fishing Lake, Kansas.	One-way ANOVA for data with normality and Kruskal–Wallis One-way ANOVA for data with failed normality using SigmaPlot.	1. Elevated tissue lead concentrations and inhibited blood ALAD enzyme activities were consistently found in birds at all contaminated sites.
18	Yoo & Janz (2003)	Determine HSP70 protein expression in head kidney, liver, gill, and ovarian tissues; and examine reproductive physiological responses in female fishes exposed chronically to sublethal metal concentrations.	Quantitative; (two fish species were collected in pre-spawning period and recrudescence period).	Season (spring and winter) and site (Tar Creek and Lytle Creek)	The 70-kDa stress protein family (HSP70) level.	*n* = not clear, Bluegill Sunfish and Black Bullhead from Tar Creek (study site) and Lytle Creek (reference site).	Two-way ANOVA and student t-tests to detect differences between reference and metal-exposed fish.	1. HSP70 expression was consistently elevated in the head kidney of both fish species collected at Tar Creek in comparison to fish collected from the reference creek. 2. In contrast, no consistent differences were observed in HSP70 expression in liver, gill, or ovarian tissues between sites
19	Schmitt et al. (2006)	Evaluate potential human and ecological risks associated with metals in fish and crayfish from mining in the TSMD.	Quantitative; (fish of six frequently consumed species collected from the Oklahoma waters of the Spring River and Neosho River).	Metals contaminations in aquatic organisms in Spring River vs. Neosho River.	Diets of Native Americans and wildlife, potential hazards of Pb, Zn, and Cd in these organisms to fish, wildfire, and humans.	*n* = varied by species (5–60 animals), fish and crayfish samples form Spring River and Neosho River.	Separate one-way ANOVA.	1. Metals concentration were typically higher in samples from sites most heavily affected by mining and lowest in reference samples. Within the TSMD, most metals concentration were higher at sites on the SR than on the NR and were typically highest in common carp and crayfish than in other taxa.

**Table 2:** Manuscripts Quality Assessment.

Studies	Objective	Design	Method	Subjects	Random	Blinding Investigations	Blinding Subjects	Measures Outcomes	Sample Size	Analytic Methods	Variance	Controlled for Confounding	Results	Conclusions	Quality Score (%)
1	2	2	1	2	N/A	N/A	N/A	2	1	2	2	2	2	2	90.9
2	2	2	2	1	N/A	N/A	N/A	2	2	2	2	2	2	2	95.4
3	2	2	2	2	N/A	N/A	N/A	2	1	2	2	2	2	2	95.4
4	2	2	2	2	N/A	N/A	N/A	2	2	2	2	2	2	2	100
5	2	2	1	2	N/A	N/A	N/A	2	2	2	2	2	2	2	95.4
6	2	2	2	2	N/A	N/A	N/A	2	2	2	2	2	2	2	100
7	2	2	1	2	N/A	N/A	N/A	2	1	2	2	2	2	2	90.9
8	2	2	1	2	0	N/A	N/A	2	2	2	2	2	2	2	87.5
9	2	2	2	2	N/A	N/A	N/A	2	2	2	0	2	2	2	90.9
10	2	2	2	2	N/A	N/A	N/A	2	2	1	2	2	2	2	95.4
11	2	2	1	2	2	N/A	N/A	2	2	2	2	2	2	2	95.8
12	2	2	1	2	2	N/A	N/A	2	2	2	2	2	2	2	95.8
13	2	2	1	2	0	N/A	N/A	2	1	2	2	2	2	2	83.3
14	2	2	2	2	N/A	N/A	N/A	2	2	2	2	2	2	2	100
15	2	2	2	2	N/A	N/A	N/A	2	1	2	2	2	2	2	95.4
16	2	2	2	2	N/A	N/A	N/A	2	1	2	2	2	2	2	95.4
17	2	2	1	1	N/A	N/A	N/A	2	1	2	2	2	2	2	86.3
18	2	2	2	2	N/A	N/A	N/A	2	1	2	2	2	2	2	95.4
19	2	2	2	2	N/A	N/A	N/A	2	2	2	2	2	2	2	100

1. Question: Questions or objective sufficiently described? 2. Design: Design evident and appropriate to answer the study question? 3. Method: Method of subject selection (and comparison group selection, if applicable) or source of information/input variables is described and appropriate? 4. Subjects: Subjects (and comparison group, if applicable) characteristics or input variables/information sufficiently described? 5. Random: If the random allocation to treatment group was possible, is it described? 6. Blinding Investigation: If interventional and blinding of investigators to the intervention was possible, is it reported? 7. Blinding Subjects: If interventional and blinding of subjects to the intervention was possible, is it reported? 8. Measure Outcome: Outcome and (if applicable) exposure measure(s) well defined and robust to measure/ misclassification bias? Means of assessment reported? 9. Sample Size: Sample size appropriate? 10. Analytic Methods: Analysis described and appropriate? 11. Variance: Some estimate of variance (e.g., confidence intervals, standard errors) is reported for the main results/outcomes? 12. Controlled for Confounding: Randomized study, with a comparability of baseline characteristics, reported. Or appropriate control at the design or analysis stage. 13. Results: Results reported in sufficient detail? 14. Conclusions: Do the results support the conclusion?
